# Mobile robotics in smart farming: current trends and applications

**DOI:** 10.3389/frai.2023.1213330

**Published:** 2023-08-31

**Authors:** Darío Fernando Yépez-Ponce, José Vicente Salcedo, Paúl D. Rosero-Montalvo, Javier Sanchis

**Affiliations:** ^1^Instituto Universitario de Automática e Informática Industrial, Universitat Politècnica de València, Valencia, Spain; ^2^Facultad de Ingeniería en Ciencias Aplicadas, Universidad Técnica del Norte, Ibarra, Ecuador; ^3^Computer Science Department, IT University of Copenhagen, Copenhagen, Denmark

**Keywords:** mobile robotics in agriculture, smart farming, path planning in agriculture, IoT in agriculture, unmanned ground vehicle in agriculture, precision agriculture, intelligent agriculture

## Abstract

In recent years, precision agriculture and smart farming have been deployed by leaps and bounds as arable land has become increasingly scarce. According to the Food and Agriculture Organization (FAO), by the year 2050, farming in the world should grow by about one-third above current levels. Therefore, farmers have intensively used fertilizers to promote crop growth and yields, which has adversely affected the nutritional improvement of foodstuffs. To address challenges related to productivity, environmental impact, food safety, crop losses, and sustainability, mobile robots in agriculture have proliferated, integrating mainly path planning and crop information gathering processes. Current agricultural robotic systems are large in size and cost because they use a computer as a server and mobile robots as clients. This article reviews the use of mobile robotics in farming to reduce costs, reduce environmental impact, and optimize harvests. The current status of mobile robotics, the technologies employed, the algorithms applied, and the relevant results obtained in smart farming are established. Finally, challenges to be faced in new smart farming techniques are also presented: environmental conditions, implementation costs, technical requirements, process automation, connectivity, and processing potential. As part of the contributions of this article, it was possible to conclude that the leading technologies for the implementation of smart farming are as follows: the Internet of Things (IoT), mobile robotics, artificial intelligence, artificial vision, multi-objective control, and big data. One technological solution that could be implemented is developing a fully autonomous, low-cost agricultural mobile robotic system that does not depend on a server.

## 1. Introduction

In recent years, the global population has increased unprecedentedly, leading to significant changes in food demand (Dhumale and Bhaskar, 2021). As we move into the future, it is expected that the demand for food will continue to rise, driven by factors such as population growth, urbanization, and changing dietary preferences. In addition, the effects of climate change have also impacted food demand and supply, creating new challenges for the food industry (Dutta et al., 2021). In Springmann et al. (2018), it is mentioned that by 2050, the food chain might increase production by 50%. Besides, the FAO shows that the world population will reach approximately 10 billion by that year (Ahmed et al., 2018). This population increase affects the environmental conditions, which changes the harvesting process forcing farmers to use fertilizers and pesticides (Shafi et al., 2019). The residuals of those chemical pollutants contaminate water (Rajeshwari et al., 2021). Another concern is the nutritional outcome that offers food since the previous statement that the environmental condition worsens, creating floods and droughts. Therefore, humans are not receiving enough nutrients to be healthy by eating processed food, requiring pills and supplements (Mostari et al., 2021). The Intergovernmental Panel on Climate Change (IPCC) warns that global warming reduces the nutritional value of crops due to the intensive use of fertilizers to boost crop yields; they also predict that in the incoming years, people might suffer from zinc deficiency, causing even their psychological and cognitive disorders (Ryan et al., 2021).

Technology in the food production industry is a significant challenge that impedes progress and innovation in this critical sector. With the rapidly growing global population and increasing demand for food, it has become imperative to adopt technological advancements to improve food production and distribution (Ferrag et al., 2021). However, in many parts of the world, particularly in developing countries, technology in food production still needs to be improved, resulting in low productivity, high food losses, and reduced efficiency. Given that a big part of food production is from developing countries, exists a lack of advanced agricultural technologies (Khan et al., 2021). They face significant financial constraints and limited access to modern technologies, which can impede their ability to improve their food production processes. This concern also extends to the education and training of the workforce, who may not have the knowledge and skills to operate and maintain technological tools and equipment effectively (Xuan, 2021).

To mitigate the concerns mentioned above about food supply, FAO proposes four bullet points to guarantee food quality in the incoming years, which they closely related to the use of technology since information plays a fundamental role in ensuring the economic and sustainability impacts of new cutting-edge techniques in the food production process (Mooney, 2020).

Implementing emerging technologies in agriculture is often called smart farming, which aims to improve productivity, efficiency, and sustainability (Raj et al., 2021). In Belhadi et al. (2021), mention that smart farming might use trend technologies such as robotics, artificial intelligence, and the IoT. Therefore, these devices can gather data from crops to extract intrinsic knowledge from plants to improve agricultural decision-making and reduce environmental impact (Megeto et al., 2021). However, the full exploitation of the potential of smart farming presents several challenges and technical, socio-economic, and administrative constraints (Mengoli et al., 2021). Works such as Ahmed et al. (2016), Jawad et al. (2017), Bermeo-Almeida et al. (2018), Kamilaris and Prenafeta-Boldu (2018), and Rahmadian and Widyartono (2020) present broad approaches to smart farming and trend technologies without focusing only on robots. These studies do not include a detailed discussion of the tools and techniques used to develop the different mobile systems and their level of maturity. It is relevant to discuss the use of mobile robotics in smart farming from different perspectives and describe their corresponding nuances.

This article stands out from others of a similar nature because it offers a broad overview of the challenges and opportunities presented by precision agriculture and robotic farming. The article focuses on the use of robotics and precision agriculture in agriculture 4.0 and provides a detailed description of the many types of agricultural robots used, as well as the techniques and hardware used for their operation and monitoring. Additionally, the article highlights the areas where literature is least developed and suggests potential solutions to address these challenges. Future trends in precision agriculture and robotics are also discussed, including the use of multi-objective control algorithms and artificial intelligence in low-cost mobile robots for planning the best path while accounting for energy efficiency, soil type, and obstacles, as well as for evaluating and managing pests and diseases that affect crops.

This work aims to present an overview of mobile robotics implemented for agricultural production related to smart farming techniques. The main contribution of this work is showing the existing frameworks, tools, and applications where robots are currently used. Also, it presents shortcomings in smart farming applications, which might provide future trends in robots. The rest of the manuscript is structured as follows: Section 2 gives the smart farming background and provides a detailed overview of the leading mobile robots with existing technologies. Section 3 presents the discussion highlighting the technical and socio-economic obstacles to successfully integrating mobile robotics in agriculture. Section 4 presents the future trends related to mobile robotics in agriculture. Finally, Section 5 presents the conclusions.

## 2. Research methodology

A systematic literature review (SLR) was performed to manage the diverse knowledge and identify research related to the raised topic (Ahmed et al., 2016), especially to investigate the status of mobile robotics in precision agriculture. In particular, we searched for papers on “mobile robotics” with the term “agriculture 4.0” in the title, abstract or keywords. Prior to the SLR, a review protocol was defined to ensure a transparent, high quality and comprehensive research process (Page et al., 2021) including three steps: formulating the research questions, defining the search strategy, and specifying the inclusion and exclusion criteria. The preferred reporting approach for systematic reviews and meta-analyses (PRISMA) was used to conduct the SLR.

### 2.1. Review protocol

Before starting the bibliographic analysis, a review protocol was defined to identify, evaluate and interpret the relevant results of the research topic (see Table 1). The first step was to formulate research questions to identify the studies published on the subject of interest from different approaches. The appropriate keywords were then identified order to formulate search strings to obtain relevant information using four databases: IEEE Xplore, Web of Science, Scopus, and ScienceDirect. To refine the search results, inclusion and exclusion criteria were defined to evaluate the content of the publications and used as a preliminary filter of the metadata sources and limit the scope of the research.

**Table 1:** Review protocol for SLR.

**Review questions**	**RQ1: How are mobile robotics used in agriculture?**

	RQ2: What technologies, methods, and tools are being used in agricultural fields?
	RQ3: What are the main challenges of multi-objective control in agriculture 4.0?
Selection criteria	**Inclusion criteria:**
	- Journal and conference articles.
	- Research published during the period from 2016 to 2022.
	- Studies that provide answers to the research questions posed.
	- Literature focused on the application of mobile robotics in agricultural field activities.
	- Early access articles.
	**Exclusion criteria:**
	- Summaries of events and seminars.
	- Literature focused on the application of mobile robotics to post-field stages.
	- Publications not available in full text.
	- Articles in languages other than English.
Literature search	**Sources:** IEEE Xplore, Web of Science, Scopus, and ScienceDirect for academic literature.
	**Search strings were used:** “agriculture” AND “UGV” AND “Path Planning” OR “Multi-objective Control” OR “Path Tracking” OR “Precision Agriculture” OR “Smart Farming” OR “Agriculture 4.0” OR “IoT”

**Figure 1:**
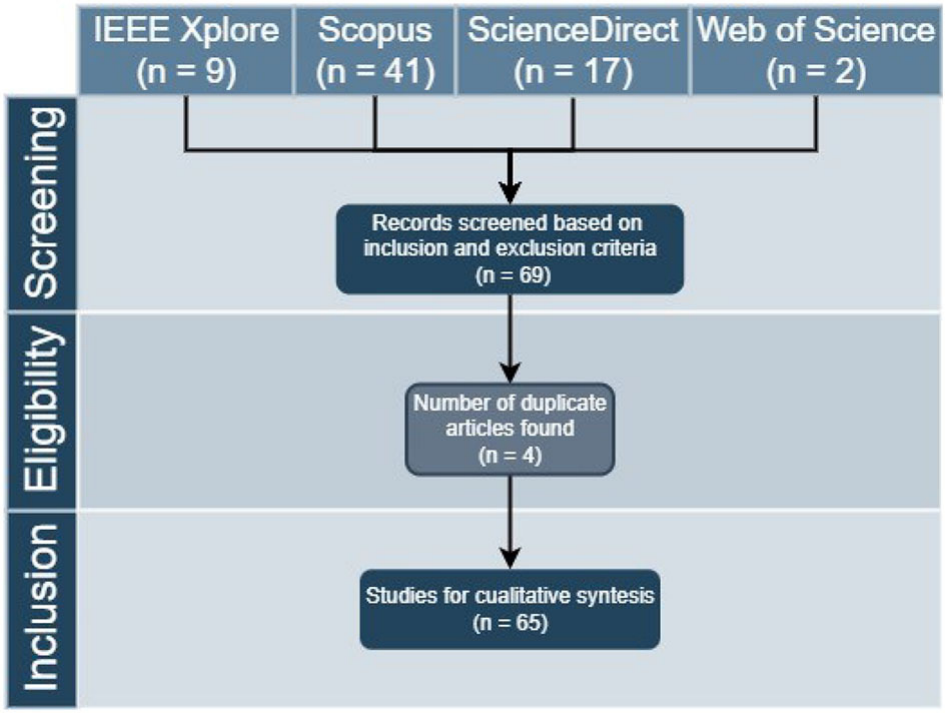
Three-steps evaluation of literature search process (PRISMA).

After performing the SLR, 69 research articles were obtained on the proposed topic. After the PRISMA selection and eligibility steps with the help of the Mendeley bibliographic reference manager, similar files were identified and eliminated, leaving a total 65 research papers, as can be seen in Figure 1.

### 2.2. Trends in agriculture

The distribution of the 65 articles by year, about 38% of the most recent scientific papers were published in 2021, reflecting the considerable progress of agriculture in the context of mobile robotics, although the pace can still be considered slow compared to other domains such as healthcare, the manufacturing, the mining, the automation, the energy, among others (Araújo et al., 2021).

**Figure 2:**
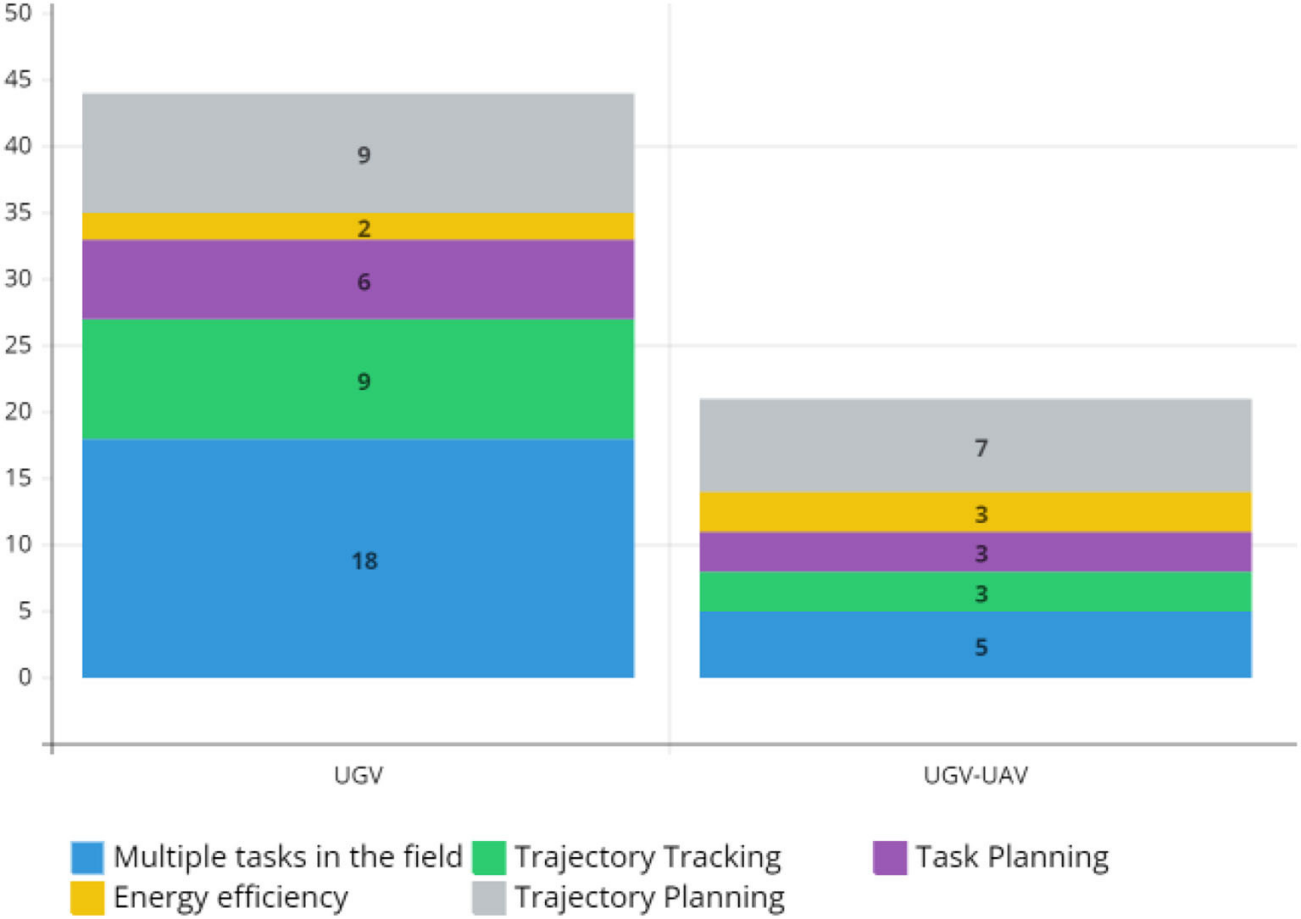
Mobile robotics activities in agriculture.

Figure 2 gives the breakdown of publications on the five most common activities carried out by agriculture 4.0 and the type of mobile robot employed. The multiple tasks in the field category include activities such as row recognition and tracking, obstacle detection and avoidance, and information gathering and reporting in both outdoor and greenhouse agriculture.

**Figure 3:**
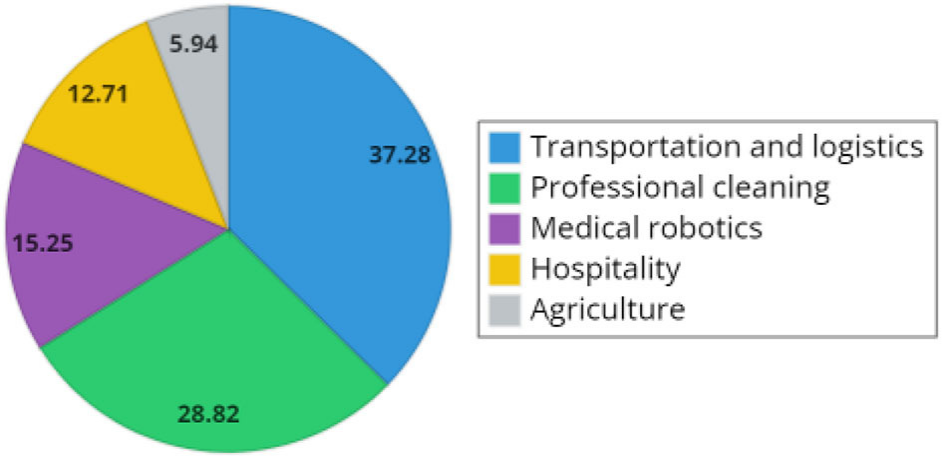
Percentage of top five applications of service robots in 2020.

According to the International Federation of Robotics (IFR), the top five service robot applications for professional use sold during 2019 and 2020 are: transportation and logistics, professional cleaning, medical robotics, hospitality, and agriculture (International Federation of Robotics, 2021). Figure 3 gives the percentage of robots employed in each of these areas.

## 3. Background and related works

Smart farming is a technique that uses advanced technology to optimize yield and efficiency in agricultural production. In Lohchab et al. (2018), explored the application of IoT technologies in smart agriculture. Subsequently, in a 2020 review article, Sharma et al. (2020) focused on the use of artificial intelligence and machine learning in smart agriculture. Furthermore, in a 2021 review article, Ratnaparkhi et al. (2020) discussed the implementation of sensor technologies and Geographic Information Systems (GIS) for smart agriculture. Finally, in a recent 2022 review article, Botta et al. (2022) examined the integration of robotics and automation in smart agriculture. Some topics that very little has been addressed in smart agriculture are: the integration of smart agriculture with the circular economy and environmental sustainability, the development and application of artificial intelligence and machine learning technologies in pest and disease identification and management, increased focus on optimizing water use and irrigation management in response to climate change and limited water availability, improved connectivity and interoperability of systems to facilitate large-scale adoption and implementation, and the development of specific low-cost solutions for small farms and rural communities in developing countries to improve food security and reduce rural poverty.

### 3.1. Smart farming

Smart farming is based on the information provided by sensors placed on an agricultural field (Ahmed et al., 2016); Machine Learning (ML) models could learn patterns to support the farmers' decision-making (Mammarella et al., 2020; Shorewala et al., 2021). These sensors joined with a microcontroller sending data constantly, are considered part of the IoT. Besides, data might be processed in big servers allocated in the cloud (cloud computing). However, IoT devices are often a rigid solution since they are placed in a single location. Therefore, Autonomous Robotic Systems (ARS) can walk around crops taking data from the whole farm and providing accurate information (Ozdogan et al., 2017; Kamilaris and Prenafeta-Boldu, 2018). This combination of sensors, data analysis, and robots provides farmers with a smart farming application with diverse tools to address challenges related to productivity, environmental impact, food safety, crop losses, and sustainability. The objectives of smart farming are to increase crop yields, minimize costs, and improve product quality through using a modern system (Araújo et al., 2021). In the last years, with technological evolution, different types of sensors have been developed that make it possible to collect data in almost any location, allowing real-time monitoring of agricultural fields without wiring. Therefore, the three leading technologies that contribute significantly to this field are as follows:

- **Drones:** These are small flying robots commonly used for crop monitoring, food infrastructure inspection, supply chain monitoring, and food safety surveillance (Costa et al., 2021).
- **Autonomous tractors:** These are generally Unmanned Ground Vehicles (UGV) incorporating sensors and actuators that enable crop monitoring, irrigation, harvesting, and disease control (Lisbinski et al., 2020).
- **Software for decision making:** These are platforms where data acquired by drones and/or UGV sensors are visualized and analyzed. They generally provide information on weather, soil, crop yields, and other factors relevant to agricultural production to improve decision-making (Ojeda-Beltran, 2022).

### 3.2. Mobile robotics in agriculture

The emerging field of agricultural mobile robotics is UGV and UAV (Prakash et al., 2020). The main applications of mobile robotics in farming are:

- Identify the state of the crop and corresponding application of chemical products, fumigation, or harvesting, as required by the fruit or plant.
- Mobile handling through collaborative arms (harvesting, fruit handling).
- Collection and conversion of helpful information for the farmer.
- Selective application of pesticides and avoidance of food waste.

UGV and UAV have limited available power. Therefore, their design and control optimization is paramount for their application in smart farming. Therefore, research on the cooperation between UGV and UAV is being carried out to cover large agricultural areas. These autonomous robots are intelligent machines capable of performing tasks, making decisions, and acting in real-time with a high degree of autonomy (Rahmadian and Widyartono, 2020). Interest in mobile robotics in agriculture has grown considerably in the last few years due to its ability to automate tasks such as planting, irrigation, fertilization, spraying, environmental monitoring, disease detection, harvesting, and weed and pest control (Araújo et al., 2021). Furthermore, mobile robotics in smart farming uses a combination of emerging technologies to improve the productivity and quality of agricultural products (Bechar and Vigneault, 2016).

UGV are robots that control can be remote (controlled by a human operator through an interface) or fully autonomous (operated without the need for a human controller based on AI technologies) (Araújo et al., 2021). The main components of UGV are locomotive, manipulator and supervisory control systems, sensors for navigation, and communication links for information exchange between devices. The main locomotion systems used are wheels, tracks, or legs. To properly operate UGV in the field, they must meet size, maneuverability, efficiency, human-friendly interface, and safety requirements. Table 2 summarizes the diverse range of UGVs designed for agricultural operations.

**Table 2:** Different types of UGV in agriculture 4.0.

**Application**	**Control**	**Hardware**	**Citation**
Fusion of color and depth images	Remote-controlled	Kinect v2 sensor and laptop computer	Gai et al., 2020
Artificial vision and weed algorithms	Remote-controlled and autonomous	AgBotII Robotic platform: RGB camera, GPS, PLC, laptop, others	Bawden et al., 2017
Grid-based using Neural Network	Autonomous	Laptop and obstacle sensor	Arindam et al., 2018
Sampling-Based Method and PSO	Autonomous	Laptop computer	Prakash et al., 2020
Non-holonomic A^*^	Autonomous	An all-terrain vehicle developed by Polaris with LiDAR, IMU, and GPS	Zhang et al., 2019
D–S evidence theory	Autonomous	Laptop, camera, and laser scanner	Zhao et al., 2019
Genetic algorithm	Autonomous	Laptop	Tsiogkas and Lane, 2018
Automated Deployment of IoT Networks	Autonomous	Pioneer 3-AT, Raspberry Pi B2, IMU, GPS, Arduino Nano, and laptop	Romeo et al., 2020
UTR control using VSC and Hyper Schemes, and Fuzzy potential motion	Autonomous	Computer	Banihani et al., 2021
Heuristic path planning	Autonomous	Computer	Wang et al., 2021
Grid-based algorithms Dijkstra, A^*^, and the sampling-based algorithms RRT and RRT^*^	Autonomous	Jackal from Clearpath Robotics with Jetson TX2, and LiDAR	Pak et al., 2022
Kalman filter estimation	Autonomous	Car-like-type, GPS, a 3D orientation sensor, and KNRm controller	Sun and Liang, 2022
Allan Variance in a Kalman Filter	-	Laptop and GPS	Luo et al., 2019
Ant Colony Optimization with a Probability − based random − walk strategy and an Adaptive waypoints	Autonomous	Laptop	Liu et al., 2022
Robust model predictive control (RMPC)	Autonomous	Husky A200 with IMU, LiDAR, laptop	Khan et al., 2022
Traveling salesman problem (TSP) enhanced with − Coverage path planning (CPP)	Autonomous	Computer	Xie and Chen, 2020
Generation optimal polynomial trajectories	Autonomous	Rover Dedalo	Gentilini et al., 2021
Compound Fuzzy Control	Autonomous	Jingguan PZ-60 with STM32 controller, laptop, and position sensors	Li et al., 2020
A^*^ Algorithm	Manual controlled	The Kinect sensor, and laptop	Nerlekar et al., 2022
Dynamic analysis of skid-steering tracked vehicles	Autonomous	Robot smaller than a standard tractor	Tazzari et al., 2020
Auto-guidance algorithm	Manual controlled	Stereo camera, angle sensor, electric power steering, GPS, and workstation ZBOX QK7P3000	Changho et al., 2021
Voronoi diagram	Autonomous	Multi-robot	Kim and Son, 2020
Algorithm row − change maneuver	Autonomous	Robotic platform with LiDAR	Mengoli et al., 2021
Machine vision algorithms	Wheels	RGB camera, and laser distance sensor	Berenstein and Edan, 2018
Harvesting	Convolutional neural networks	Platform Vegebot with camera, UR10 controller, and laptop	Birrell et al., 2020
Smart irrigation	Teleoperation	NodeMCU, water level sensor, Arduino, temperature and humidity sensor	Srinivas and Sangeetha, 2021

The main issue of mobile robotics in agricultural fields is to perform multiple tasks (obstacle avoidance, tracking, path planning, crop data collection, disease detection, among others) autonomously with reduced hardware for low-cost robots that can be acquired and implemented by farmers. Most UGV presented above have a wheeled locomotion system, offering easy construction and control. Some UGV incorporate low-cost computer vision systems, i.e., using conventional cameras. UGV might employ heuristic algorithms still in the conceptual or prototyping phase. Due to the limitations of UGV and to cover larger areas and less time, in the last years, the UGV-UAV collaboration has been developed (Khanna et al., 2015). The UGV operates in the areas selected by the UAV, which also cooperates in the generation of 3D maps of the environment with centimeter accuracy; however, merging the maps generated by UAV and UGV in an agricultural climate is a complex task since the generated maps present inaccuracies and scale errors due to local inconsistencies, missing data, occlusions, and global deformations (Gawel et al., 2017; Potena et al., 2019). Table 3 reviews some collaborations between UGV and UAV in smart farming.

**Table 3:** Collaborations between UGV and UAV in smart farming.

**Primary function**	**Techniques used**	**Hardware**	**Citation**
Monitoring and drone landing	Re-configurable chassis	Solar panels	Quaglia et al., 2018
Optimal control for the refueling of UAV	Non-linear optimal control	Computer	Rucco et al., 2017
UAV/UGV trajectory planning	Partial Differential Equation (PDE)	Computer	Radmanesh et al., 2021
	Energy-constrained minimization	Computer	Edmonds et al., 2021
Path-following of UGV/UAV	Null Space control	Computer	Bacheti et al., 2021
Ground station for UAV/UGV	Improved Ant Colony algorithm	Commercial UAV/UGV, communication module, and Computer	Liang et al., 2021

Most collaborative systems between UAV and UGV are in the conceptual (simulation) phase.

### 3.3. Multi-objective control in smart farming

Agricultural systems use and produce energy in the form of bioenergy and play a vital role in the global economy and food security. Modern agricultural systems might therefore consider economic, energy, and environmental factors simultaneously (Banasik et al., 2017). Multi-objective control is an important tool in smart farming to simultaneously run and optimize multiple objectives, such as productivity, water use efficiency, product quality, and economic profitability. Some cases of multi-objective control in smart farming are presented in Table 4, which shows their primary function, control techniques, and the hardware deployed. However, there are few studies since this topic is new in smart farming applications with mobile robots. Furthermore, path planning is an essential application of smart agriculture that focuses on optimizing routes and movements of agricultural machinery to improve efficiency and reduce production costs (Nazarahari et al., 2019).

**Table 4:** Multi-objective control in agriculture 4.0.

**Primary function**	**Techniques used**	**Hardware**	**Citation**
Energy management	Multi-objective genetic algorithm (MOGA)	Computer	Shamshirband et al., 2015
Crop pattern optimization	Crow Search Algorithm (CSA) and Particle Swarm Optimization (PSO)	Computer	Jain et al., 2021
Optimization of water-food-energy	Fuzzy multi-objective programming model	Computer	Li et al., 2019
Path planner	Non-dominated Sorting Genetic Algorithm using Reference Point Based (NSGA-III)	Computer	Azimi-Mahmud et al., 2019
	MP-PSOGA algorithm	Computer, and UAV	Zhai et al., 2018
	Combinatorial multi-objective	MGV and UGV	Chirala et al., 2021
	Multi-objective particle swarm optimization (CMOPSO)	Computer	Mac et al., 2017
Irrigation	General Algebraic Modeling System (GAMS)	Computer	Galán-Martín et al., 2017

Another application of multi-objective control in path planning is the optimization of fertilization and pesticide application in crops. According to a study by Zhao et al. (2023), multi-objective control can optimize the routing of pesticide and fertilizer application machinery to reduce the number of inputs used and improve application efficiency. In addition, multi-target control can also improve product quality and reduce environmental pollution by accurately applying crop inputs.

Finally, a study proposes a Residual-like Soft Actor-Critic (R-SAC) algorithm for agricultural scenarios to realize safe obstacle avoidance and intelligent path planning of robots. The study also proposes an offline expert experience pre-training method to improve the training efficiency of reinforcement learning. Experiments verify that this method has stable performance in static and dynamic obstacle environments and is superior to other reinforcement learning algorithms (Yang et al., 2022).

## 4. Discussion

With the information mentioned above about mobile robots in smart farming, this section aims to show the future steps in this research field related to its challenges. Given the new UGV and UAV trends in Table 2, the multi-objective control has yet to be widely explored in smart farming applications. It might be due to its complex setup and the expensive computational resources needed. However, multi-objective applications might be doable in incoming robots with the increasing microcontrollers and microprocessor development. Conversely, IoT devices that collect data from farms are extensively deployed in several applications. However, there are new concerns about their confidentiality and the risk that data is exposed when traveling by communication channels (Pylianidis et al., 2021).

Smart farming needs final devices with robust systems working in harsh conditions in outdoor scenarios. However, several works have shown prototypes with their tentative functionalities. Building robots may need several debugging rounds to solve issues with the hardware and software. Consequently, since the robot links people and plants, farmers, considered experts in smart farming, must work closely with the robot's developer. However, the variety of plant and crop species makes it challenging to develop a multi-task robot (Selmani et al., 2019).

The main challenges and future research for deploying smart farming are presented. The present study sought to articulate mobile robotics with smart farming. Looking at Table 4, it can be seen that multi-objective control has not been significantly explored in smart farming. One of the reasons could be that applying advanced technologies with complex operations can be costly. Hence, the development of these technologies in smart farming should increase in the coming years. Also, the IoT is widely deployed in agriculture for crop monitoring and tracking. Therefore, it can be said that IoT is a research trend within smart farming. However, only a few studies have considered data security and reliability, scalability, and interoperability when developing a smart farming system (Pylianidis et al., 2021).

The results presented also show that most of the use cases are in the prototype phase. One possible reason could be that smart farming links people, animals, and plants making it more difficult than creating systems for non-living things. Another reason could be that the technology is due to the transdisciplinarity of this field, and therefore for the development of intelligent systems, farmers should be familiar with these technologies. Finally, the variety of plant and crop species makes implementing technology in agricultural fields complex (Selmani et al., 2019). The results also show that most systems developed are for free-range farms. In addition, it is also evident that research is limited to soil management, fruit detection, and crop quality management. With this, it is corroborated that work must be done on research and development of systems that guarantee the deployment of smart farming at affordable costs. The natural complexity of agricultural fields presents a number of obstacles that prevent the full integration of mobile robotics in smart farming. Therefore, from the analysis, blockages at the technical and socio-economic levels have been identified and classified.

### 4.1. Technical roadblocks

- **Interoperability**. To establish effective communication between heterogeneous devices, they need to be interconnected, and interoperable (Aydin and Aydin, 2020).
- **Dataquality**. Lack of decentralized systems impedes the deployment of smart farming (Liu et al., 2022).
- **Hardware**. A suitable casing must be constructed that is robust and durable enough to withstand actual field conditions (Villa-Henriksen et al., 2020).
- **Power sources**. A proper energy-saving scheme is necessary as instant battery replacement is complicated. A possible solution to optimize power consumption is using low-power hardware and proper communications management (Jawad et al., 2017).
- **Wireless architectures**. Wireless communication networks and technologies offer several advantages in terms of low cost, wide area coverage, network flexibility, and high scalability (Brinis and Saidane, 2016).
- **Security**. The nature of agricultural fields leads to risks to data privacy, integrity, and availability (Chen et al., 2017).
- **User interface**. Most graphical user interfaces are designed so that only experts can use them (Del Cerro et al., 2021).

### 4.2. Socio-economic roadblocks

- **Costs**. Costs associated with adopting robotic technologies and systems are the biggest drawback to deploying smart farming (Sinha and Dhanalakshmi, 2022).
- **Return on investment**. When implementing new technologies, farmers are concerned about the payback time and the difficulties in assessing the benefits (Miranda et al., 2019).
- **Gap between farmers and researchers**. Farmer involvement is paramount to the success of smart farming. Farmers face many problems during the production process that technology could solve (Bacco et al., 2019).

Finally, in Charatsari et al. (2022) discusses the importance of responsibility in the process of technological innovation in the agrifood industry. It highlights the need to consider not only technical aspects but also social implications and societal values when introducing innovative technologies. The authors argue that the perception of responsible innovation is limited in various industrial sectors, making it challenging to implement responsible innovation approaches. The complexity of responsible innovation in the agrifood industry requires addressing the multiple scales and levels of interaction between actors and the constant evolution of agrifood systems. Therefore, the article emphasizes the need to adopt responsible innovation practices that consider the social, ethical, and environmental implications of technological innovations in the industry.

## 5. Future trends

The upcoming initiatives related to using robots represent significant improvements in smart farming. Government initiatives, public-private sectors, and research work in this field might contribute to establishing the right conditions to add new hardware to crops. However, there are some challenges to consider when developing mobile robots in agriculture such as: navigation on uneven terrain (loose soil and unpredictable obstacles) without damaging plants or compromising their own safety, energy efficiency so that they can operate for long periods of time avoiding constant human intervention, crop manipulation, integration with farm management systems and adaptability to different crops and conditions.

For instance, a robotic system can be developed for smart farming, starting from a basic architecture with few components and simple functionality that allows the gradual addition of features and functionality to create a complex system. Future trends in smart farming involve using multi-objective control algorithms and artificial intelligence in low-cost mobile robots to plan the best trajectory considering energy efficiency, soil type, and obstacles while monitoring crop growth and assessing and controlling crop pests and diseases. To ensure good connectivity and live transmission of crop data, 5G technology needs to be widely explored. 5G technology minimizes internet costs and increases information management by remotely performing accurate inspections of agricultural fields (Abbasi et al., 2021). Finally, blockchain, combined with IoT and other technologies, should be applied to address the challenge of information privacy and security (Bermeo-Almeida et al., 2018).

As seen in the tables in the previous sections, most of the UGV have a computer, which increases the cost of this type of robots. Table 5 shows several state-of-the-art boards that could deploy smart farming at affordable prices for farmers.

**Table 5:** Boards for agriculture 4.0.

**Feature**	**Description**
Raspberry Pi 4	Model: B+
	System on a chip: Broadcom BCM2711
	CPU:1.5 GHz quad-core processor with Cortex-A72 arm
	GPU: VideoCore VI
	Memory: 1/2/4GB LPDDR4 RAM
	Connectivity: 802.11ac Wi-Fi / Bluetooth 5.0, Gigabit Ethernet
	Video and sound: 2 x micro-HDMI ports supporting 4K@60Hz displays via HDMI 2.0, MIPI DSI display port, MIPI CSI camera port, 4-pole stereo output and composite video port.
	Ports: 2 x USB 3.0, 2 x USB 2.0
	Power Supply: 5V/3A via USB-C, 5V via GPIO header
	Expansion: 40-pin GPIO header
Jetson Nano	Model: B01
	CPU:ARM A57 quad-core
	GPU: NVIDIA Maxwell 128-core
	RAM: 4 GB of 64-bit LPDDR4
	Video Encode: 4K @ 30 | 4x 1080p @ 30 | 9x 720p @ 30 (H.264/H.265)
	Video Decode: 4K @ 60 | 2x 4K @ 30 | 8x 1080p @ 30 | 18x 720p @ 30 (H.264/H.265)
	Connectivity: Gigabit Ethernet 10/100/1000
	Power Supply: Micro-USB 5V 2A, Barrel connector 5V 4A
	Inputs and Outputs: 4x USB 3.0, USB 2.0 Micro-B, HDMI/DisplayPort, GPIO, I2C, I2S, SPI, UART, Two MIPI-CSI camera connectors, Fan connector, PoE connector.
Arduino Portenta	Model: H7
	Microcontroller:STM32H747XI Dual Cortex^®^-M7 + M4 32bit low-power Arm^®^ MCU
	Radio module: Murata 1DX dual WiFi 802.11b / g / n 65 Mbps y Bluetooth 5.1 BR / EDR / LE
	Secure element: NXP SE0502
	Power supply board (USB/VIN): 5 V
	Compatible battery: Single cell Li-Po, 3.7 V, 700 mAh minimum
	Circuit operating voltage: 3.3 V
	Consumption: 2.95 μA in standby mode
	Display connector: MIPI DSI and MIPI D-PHY host for interfacing with a large low pin count display
	GPU:Chrom-ART Graphics Hardware Accelerator^*TM*^
	Timers: 22x timers and watchdogs
	UART: 4 ports (2 with flow control)
	PHY Ethernet: 10/100 Mbps (through expansion port only)
	SD card: Interface for SD card connector (only through expansion port)
	Operational temperature: −40 °C to +85 °C
	High density connectors: Two 80-pin connectors will expose all on-board peripherals to other devices
	Camera interface: 8 bits, up to 80 MHz
	ADC: 3 × ADC with 16-bit max. resolution (up to 36 channels, up to 3.6 MSPS)
	DAC: 2 × 12 bits (1 MHz)
	USB-C: Host/device, DisplayPort output, high/full speed, power supply

**Figure 4:**
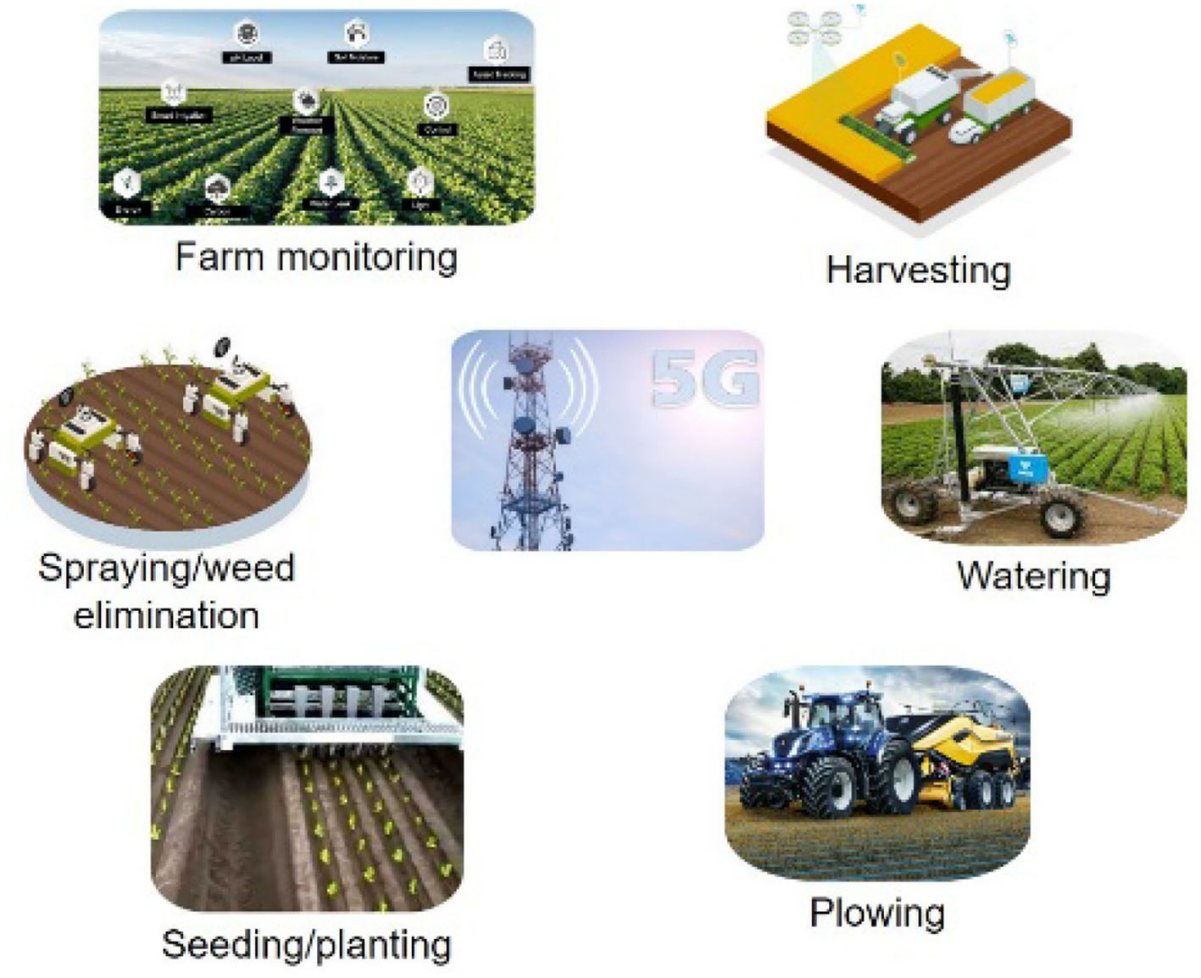
Future of agriculture.

Finally, in Figure 4, we can see the future of agriculture, for which a correct 5G network deployment and path planning/tracking is essential. Artificial intelligence, machine learning, machine vision, IoT, and cloud computing are needed in each of the activities carried out in agricultural fields.

## 6. Conclusions

Growing concerns about global food security have accelerated the need to incorporate mobile robots in agriculture. The scientific community and researchers are integrating disruptive technologies into conventional agricultural systems to increase crop quality and yields, minimize costs, and reduce waste generation. This article analyzes the current state and challenges of smart farming. Considering the impact of farming on climate change and healthy food production, it is vital to provide the agricultural sector with low-cost, functional mobile robots. Research questions were posed and answered regarding the use of mobile robotics in agriculture, the technologies, methods, and tools used in agricultural fields, and the main challenges of multi-target control in this area. Several conclusions were drawn, such as the integration of scalable mobile robots incorporating efficient systems. It should be noted that most cases address a specific problem and are in the prototype phase.

From the SLR conducted, it was identified that research on the following topics is limited:

- The implementation of digital twins for robot-based production lines
- Ingenious software project management while narrowing the impact aspect.
- Blockchain in agriculture.
- Context-aware wireless sensor network suitable for precision agriculture.
- Internet of Things (IoT) for smart precision agriculture and farming in rural areas.
- Semantic and syntactic interoperability for agricultural open-data platforms in the context of IoT using crop-specific trait.
- Multi-objective path planner for an agricultural mobile robot in a virtual and real greenhouse environment.
- Closing loops in agricultural supply chains using multi-objective optimization.
- New control approaches for trajectory tracking and motion planning of unmanned tracked robots.

These areas require further research to improve the efficiency and effectiveness of precision agriculture. Likewise, the information gathered in this article makes it clear that the emerging fields of research are:

- **Autonomous navigation**. Planning, tracking of trajectories, and task planning should be considered in this area.
- **Energy efficiency**. Good navigation autonomy is not the only thing that must be taken into account, but also the design and all components that make up the mobile robot since its size and cost directly influence the deployment of smart farming.
- **Communication**. Due to the number of devices involved in smart farming, middleware that improves communication between field devices and the station is important to ensure the reliability and security of information.

The interdependence of these challenges means that a practical solution must be sought with a suitable compromise between the theoretically optimal path that facilitates information exchange and overall system energy optimization. Moreover, the following questions must be considered: the kinematic and dynamic design of the mobile robot, the terrain traversability, the computational complexity of the various algorithms to ensure real-time performance, the use of sensors and low-energy control boards, and the sending and receiving of information. It also identifies the leading technical and socioeconomic obstacles that must be overcome to deploy smart farming successfully. We can see leaps and bounds being made in this area, but there is still a long way to go to mitigate the impact of farming on the environment in the coming years. Finally, one of the areas to be investigated is multi-objective heuristic optimization for autonomous navigation, communication, and energy efficiency of mobile robots.

Finally, numerous international political organizations play a crucial role in spreading awareness of the technologies involved in precision agriculture and advocating for their successful implementation. These organizations are:

- The FAO promotes the use of advanced agricultural technologies through programs and projects, providing technical assistance, training, and resource access for farmers.
- The European Union (EU) supports agricultural modernization and the adoption of innovative technologies in the industry through its Agricultural Common Policy (ACP). Additionally, the UE funds research and development projects in precision agriculture, agricultural robotics, and digital solutions to increase efficiency and sustainability.
- The Department of Agriculture (USDA) of the United States places emphasis on the adoption of cutting-edge agricultural technologies. The USDA supports the implementation of precise agriculture systems, the integration of sensors and IoT devices into agricultural operations, and the promotion of digitalization in the industry through its funding and grant program.
- The focus of AGRA is to encourage the use of contemporary agricultural technologies across the African continent. AGRA works in close partnership with governments, regional organizations, and the private sector to increase access to and availability of improved seeds, fertilizers, and digital farming technologies that boost agricultural productivity and sustainability.
- The World Economic Forum (WEF) has established initiatives and projects to advance precision agriculture. The WEF brings together many actors-including political leaders, business executives, and members of civil society-through its platform “Shaping the Future of Food Security and Agriculture” to develop innovative and collaborative solutions that foster the digital transformation of agriculture.

These political organizations play a crucial role in the spread of advanced agricultural technologies, and they are actively working to promote the adoption of “agriculture 4.0” on a global scale with the aim of enhancing the efficiency, productivity, and sustainability of the agricultural sector.

## Author contributions

JVS supervised this project. PR-M and JS contributed in this project. DY-P made the first version of the article under the guidelines of the other authors; likewise, he made the corrections to the observations made by the reviewers and shared them with the other authors for their respective review and subsequent approval. All authors contributed to the article and approved the submitted version.
